# Evaluating the safety profile of connectome-based repetitive transcranial magnetic stimulation

**DOI:** 10.1017/neu.2025.9

**Published:** 2025-03-21

**Authors:** Si Jie Tang, Jonas Holle, Emil Gabrielsson, Nicholas B. Dadario, Mark Ryan, Maurice Sholas, Michael E. Sughrue, Charles Teo, Jacky Yeung

**Affiliations:** 1 School of Medicine, University of California Davis Medical Center, Sacramento, CA, USA; 2 Cingulum Health, Sydney, Australia; 3 Robert Wood Johnson Medical School, Rutgers University, New Brunswick, NJ, USA; 4 Sholas Medical Consulting New Orleans, LA, USA; 5 Department of Neurosurgery, Yale University School of Medicine PO Box, New Haven, CT, USA

**Keywords:** TBS, rTMS, Connectome, Safety, Human Connectome Project

## Abstract

**Objective:**

New developments in neuro-navigation and machine learning have allowed for personalised approaches to repetitive transcranial magnetic stimulation (rTMS) to treat various neuropsychiatric disorders. One specific approach, known as the cingulum framework, identifies individualised brain parcellations from resting state fMRI based on a machine-learning algorithm. Theta burst stimulation, a more rapid form of rTMS, is then delivered for 25 sessions, 5 per day, over 5 days consecutively or spaced out over 10 days. Preliminary studies have documented this approach for various neurological and psychiatric ailments. However, the safety and tolerability of this approach are unclear.

**Methods:**

We performed a retrospective study on 165 unique patients (202 target sets) treated with this personalised approach between January 2020 and December 2023.

**Results:**

Common side effects included fatigue (102/202, 50%), local muscle twitching (89/202, 43%), headaches (49/202, 23%), and discomfort (31/202, 17%), all transient. The top 10 unique parcellations commonly found in the target sets included L8av (52%), LPGs (28%), LTe1m (21%), RTe1m (18%), LPFM (17%), Ls6–8 (13%), Rs6–8 (9%), L46 (7%), L1 (6%), and L6v (6%). Fatigue was most common in target sets that contained R6v (6/6, 100%) and L8c (5/5, 100%). Muscle twitches were most common in target sets that contained RTGv (5/5, 100%) and LTGv (4/4, 100%).

**Conclusion:**

These side effects were all transient and well-tolerated. No serious side effects were recorded. Results suggested that individualised, connectome-guided rTMS is safe and contain side-effect profiles similar to other TMS approaches reported in the literature.

Significant outcomes

1. Parcel-guided rTMS is safe with no adverse, long-lasting side effects with the most common side effects as fatigue 50% and local muscle twitching 43%.
2. No adverse side effects were reported when targeting outside of the dorsolateral prefrontal cortex.
3. No adverse side effects were reported in patients with craniotomies and strokes.

Limitations

1. There are no control or placebo group in this study
2. This is only a one site study, and differences in how clinics administer TMS, such as the choice of the geometry of the coil or what percentage of minimum effective stimulation intensity is used for TMS, may affect the side effects experienced by patients.
3. Target sets are different combinations of 3 HCP parcellations, so one cannot directly attribute side effects to one parcellation, only its target set.

## Introduction

Transcranial magnetic stimulation (TMS) has been applied therapeutically across a wide range of neurological and neuropsychiatric illnesses. It utilises Faraday’s law of electromagnetic induction, wherein a magnetic coil non-invasively generates an electric field in targeted brain tissue to cause neuron depolarisation (Hallett, 2000). When TMS is applied repetitively, it can modulate and cause lasting changes in the cortical excitability of targeted areas, exhibiting its potential for use in the treatment and management of psychiatric and neurological disorders (Klomjai *et al*., 2015). Repetitive TMS (rTMS) is a protocol applied to the dorsolateral prefrontal cortex (dlPFC) to treat medically resistant MDD, which gained FDA approval in 2008 (Cohen *et al*., 2022). Since then, rTMS and other expedited TMS protocols have been investigated for their utility across a range of psychiatric and neurological conditions including schizophrenia, bipolar disorder, post-traumatic stress disorder, tinnitus, Parkinson’s, chronic pain, and migraine (Rossi *et al*., 2021).

TMS is widely considered to be a safe and well tolerated treatment (Taylor *et al*., 2018). Common side effects include mild and include neck pain, discomfort at the stimulation site, and headache (Loo *et al*., 2008). Variability in the occurrence of these outcomes can be attributed to factors such as the stimulation intensity, frequency of pulse, and duration of treatment (Loo *et al*., 2008). Serious adverse events such as hearing impairment and affective switch have been reported, despite being exceedingly rare. Perhaps the most pertinent risk of TMS is that of seizure, and though estimates vary, the standardised risk of seizure for rTMS was approximated by Rossi et al., to be 1/100,000 and 67/100,000 (sessions) in patients without and with risk factors for seizure, respectively. Risk factors include epilepsy, neurological conditions causing structural damage to the brain, neurodegenerative diseases, meningoencephalitis, intracerebral abscesses, and cancers affecting the brain parenchyma or leptomeninges (Rossi *et al*., 2021).

Theta burst stimulation (TBS) is an expedited form of the rTMS protocol, delivering 50 Hz pulses in bursts with 5 Hz intervals. It has two forms: intermittent TBS (iTBS) and continuous TBS (cTBS) which elicit excitatory and inhibitory responses in cortical excitability respectively (Staubli and Lynch, 1987, Stoby *et al*., 2022). TBS offers shorter stimulation durations which significantly reduces patient time commitment. Traditional TMS and TBS protocols have been shown to have similar safety profiles (Lan *et al*., 2023), with TBS showing a comparable or lower risk of adverse events compared to high frequency rTMS protocols (Oberman *et al*., 2011).

The majority of rTMS research has focused on targeting areas within the dlPFC. As such, there are limited studies examining the safety and tolerability of rTMS, let alone TBS, outside of this region. Recent advances in TMS administration have allowed for neuronavigated approaches that enable targeting outside of the dlPFC to treat specific deficits (Schonfeldt-Lecuona *et al*., 2010). Early evidence suggests that the safety and tolerability profiles of targeting outside of the dlPFC may be akin to standard rTMS applied by conventional means. A review by Machii et al., examined the use of rTMS in non-motor cortical areas, thereby including the dlPFC, but also the frontal, parietal, occipital, temporal, and cerebellar areas. Similar to dlPFC targeting, the most common side effects identified were headache and neck pain, which occurred in over 40% of patients (Machii *et al*., 2006).

The Cingulum Framework is an approach which utilises connectomics to deliver personalised TMS. Connectomics is a field dedicated to comprehensively mapping the structural and functional connections of the brain. While various imaging technologies can be used to model connectomes, resting state functional magnetic resonance imaging (rsfMRI) (Smith *et al*., 2013) allows researchers to study the complex dynamics of large scale brain networks (Thomas Yeo *et al*., 2011). Dysfunction within large-scale brain networks, like the default (DMN), central executive (CEN) and salience (SN) networks, manifests as aberrant functional connectivity and has been implicated in various psychiatric and neurological conditions and their subtypes (Menon, 2011, Nicholson *et al*., 2020, Zheng *et al*., 2015, Bertocci *et al*., 2023, Fan *et al*., 2017, Young *et al*., 2023a). Importantly, TMS has been shown to successfully modulate resting state functional connectivity across the temporal, parietal, occipital and cerebellar regions (Kirkovski *et al*., 2023). As such, areas of anomalous connectivity, often outside of the dlPFC, implicated in specific conditions offer potential targets for personalised connectome guided TMS.

A recent development in AI-driven neuroimaging has led to a connectomic software (Omniscient Neurotechnology, Sydney) that utilises rsfMRI to image functional connectivity and compare subject’s brains with a dataset of 200 healthy individuals’ connectomic data from the OpenNeuro (https://openneuro.org/) and SchizConnect (http://schizconnect.org) datasets. These patients report no history of psychiatric or neurological illness. The software analyses the functional connectivity of key networks implicated in patients’ symptoms and conditions to identify regions within these networks of hyper/hypo-connectivity that may be potential target locations for cTBS or iTBS respectively. The conjunction of the Cingulum Framework and improved spatial resolution in neuronavigation has allowed us to target brain parcellations outlined by the Human Connectome Project (Glasser *et al*., 2016). It has enabled the potential for a personalised connectomic approach to the rTMS treatment of psychiatric and neurological conditions. Preliminary results of this approach have been documented in patients with major depressive disorder (MDD), generalised anxiety disorder (GAD), individuals with post-tumor craniotomies, post-concussive syndrome, and one case study of a patient following a stroke (Tang *et al*., 2023, Young *et al*., 2023b, Tang *et al*., 2022, Yeung *et al*., 2021).

Personalising treatment by analysing and targeting aberrant connectivity in large-scale distributed networks yields TMS targets across the whole brain. This approach has the potential to optimise the established treatment of conditions using TMS and offer new treatment options for under-researched and under-treated disease states. However, this nascent field of TMS application currently faces a lack of safety and tolerability studies. This study aims not to explore the efficacy of a connectome-guided approach to TMS but to document its safety and tolerability to support further trials. As such, we present a retrospective study on the safety associated with personalised connectome-guided TBS in 165 patients with various psychiatric and neurological conditions across 92 unique parcellations.

## Materials and methods

### Participants

Patients (*n* = 165) were included in this retrospective study if they completed at least one full course of connectome-guided rTMS at Cingulum Health in the period from January 2019 to December 2023. Patients were excluded from completing the course of stimulation if they could not complete a rsfMRI study. Likewise, they were excluded if they had any contraindications for rTMS, including an epilepsy diagnosis, ferromagnetic metal in the head or neck or a deep brain stimulation (DBS) device (McClintock *et al*., 2018). Patients included in this study have a range of neurological and psychiatric diagnoses (Table 1 and Supplement 1). Patients who had complex symptoms but lacked a formal diagnosis were also included. Patient data was analysed retrospectively with prospectively collected data. This study was approved by the Human Research Ethics Committee of the South Eastern Sydney Local Health District (2022/ETH00139).

**Table 1.** Patient demographics. *Patients may have multiple diagnoses. Only the most common diagnoses are included in this table. Please see all diagnoses in supplement 1

Characteristics	N (%) or *N* ± Standard Deviation *N* = 165 Total Patients 202 Total Target Scans
**Age**	46.9 ± 17.1 (range: 14 years - 87 years old)
**Sex**	
Female	69 (42)
Male	96 (59)
Diagnoses*	
Generalized Anxiety Disorder	65 (39)
Major Depressive Disorder	58 (35)
Traumatic Brain Injury	26 (16)
Cognitive Complaints (Including Alzheimer’s Disease)	23 (14)
Post-surgical Rehabilitation	15 (9)
Stroke	11 (7)
Migraine/Headaches	11 (7)

### Neuroimaging protocol

All patients prior to rTMS treatment completed a resting state fMRI (rsfMRI) and non-contrast T1-weighted MRI on a Phillips 3T Achieva. The rsfMRI was obtained as a T2-star echo-planar imaging sequence with 3 × 3 × 3-mm voxels, 128 volumes per run, a TE of 27 ms, a TR of 2.8 s, a 256 mm field of view, a 90° flip angle, and a total run time of 8 minutes. For T1-weighted 3D volume acquisition, 1-mm slices were collected with no overlap between slices. The field of view covered the entire head, achieving isotropic imaging with a 256 × 256 matrix.

### Connectomic analysis

Neuroimaging data was subsequently processed by the Omniscient Infinitome software (Sydney, Australia) as previously described (Yeung *et al*., 2021, Young *et al*., 2023b, Tang *et al*., 2023, Poologaindran *et al*., 2022, Dadario *et al*., 2022). The Infinitome software is utilised to create a parcellation of patients’ grey matter into 377 unique parcels as defined in the Human Connectome Project Multi-Modal Parcellation version 1.0 (HCP) atlas (Glasser *et al*., 2016). Outlier detection from the pairwise correlations of each parcel, a total of 142,129 values, used a tangent space functional correlation matrix, comparing results to 200 healthy rsfMRI control samples. A tangent space connectivity transformation established normal correlation ranges based on the normative atlas of healthy individuals. Abnormal connectivity was identified as a 3-sigma outlier from this normative atlas, excluding the highest variance 1/3 of pairs to reduce false discoveries, as documented previously (Young *et al*., 2023b, Tang *et al*., 2022, Tang *et al*., 2023, Dadario *et al*., 2023). The 3 standard deviation threshold ensures that identified outliers fall well beyond the normal range, capturing only those with significantly abnormal connectivity. Parcel pair connectivity is visualised through connectivity matrices in which parcels can be grouped according to their anatomical location, or according to their membership within specific brain circuits and large-scale brain networks.

**Figure 1.**
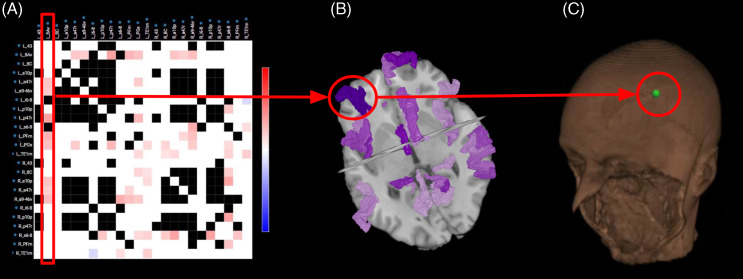
TMS target selection. (a) The anomaly matrix filters the individuals’ functional connectivity data and compares it to the control dataset (n = 200) to identify areas of anomalous connectivity within large-scale networks relevant to the patients’ symptoms. b) these anomalous regions can be visualised within the brain to ensure they are not deeper than the penetration depth of the TMS coil and are then exported to the neuro-navigation system. c) the T1 images and target area files are uploaded to the neuro-navigation system to select coil placement positions and ensure precise stimulation.

Anomalous functional connectivity is represented within the software in the form of Anomaly Matrices (Fig. 1a). Each selected parcel is displayed in a symmetrical matrix and the degree of their connectivity to another parcellation is represented according to a red, blue, white, and black key. Hyperconnected pairs in the connectivity matrix are represented in red, and hypoconnected pairs are represented in blue. These are defined based on 3 standard deviations beyond the normal range of connectivity from healthy controls as described above. Areas within the normal range of correlation are represented in white, and connections represented as black display too much signal noise in the healthy population to determine a normal range.

### Personalised target selection

Each patient was approached as an individual case, and no standardised targets were prescribed for specific conditions. The predominant approach to target selection was to analyse the functional connectivity of each patient according to the large-scale brain networks relevant to their condition or constellation of symptoms. This involved a variety of hypotheses for different clinical presentations. For example, we began with the hypothesis that anomalous functional connectivity within the Default Mode Network (DMN), Central Executive Network (CEN) and the Salience Network were associated with the symptoms of several psychiatric illnesses (Menon, 2011, Dadario, 2022). Similarly, if patients suffered functional deficits following stroke or neurosurgery, the analysis for target selection would begin with the Sensorimotor Network.

In complex cases where patients had more complex cognitive deficits, the Dorsal and Ventral Attentional Networks (DAN, VAN) were also investigated. No subjective, conscious guidance was given for the target selection for any patient. The kind and number of anomalous connectivity pairs within key networks determined the selection of either continuous theta burst (cTBS) or intermittent theta burst (iTBS) protocols.

Parcels with the most hyperconnected pairs within given networks received cTBS to induce cortical depression and conversely those with significant hypoconnected regions received iTBS (Huang *et al*., 2005).

**Figure 2.**
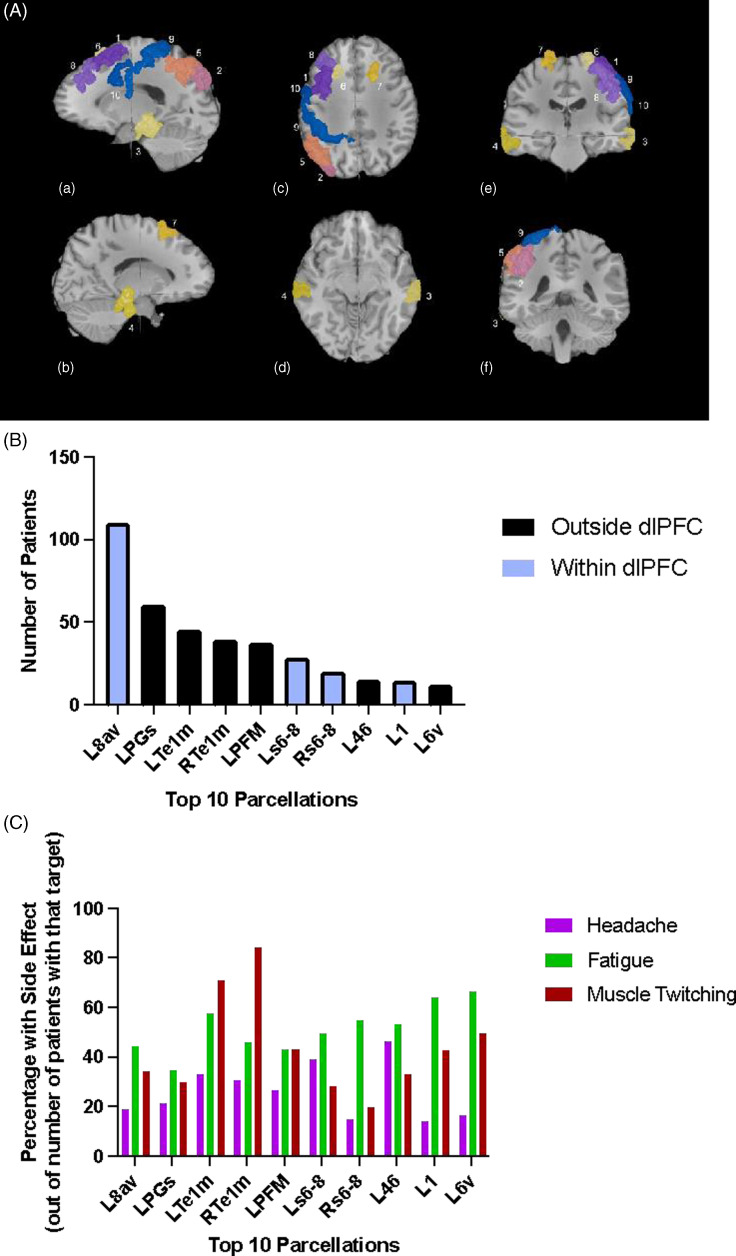
Side effect profile of the top ten stimulated parcellations. (A) locations of the top 10 most common targets in order of frequency overlaid on standardized anatomical images: 1. L8av, 2. LPGs, 3. LTe1m, 4. RTe1m, 5. LPFM, 6. Ls6-8, 7. Rs6-8, 8. L46, 9. L1 and 10. L6v. a) left sagittal, b) right sagittal, c) top axial, d) bottom axial, e) front coronal, f) back coronal. (B) histogram of the number of patients with targets in the top ten parcellations. Black bars represent parcellations outside of the dlPFC. Blue bars represent parcellations within the dlPFC. (C) histogram of the side effects experienced by patients in with targets in the top ten parcellations as a percentage of the total number of patients (from (A)) with that specific target. Pink bars represent percentage with headaches, green bars represent percentage with fatigue, and red bars represent percentage with local muscle twitching.

The overarching hypothesis in connectomic network-based target selection is that normalising the most anomalous hubs within relevant key networks is associated with symptom improvement. This approach is here referred to as ‘connectomic’ or ‘parcel-guided’ TMS as previously reported by our group and other authors (Moreno-Ortega *et al*., 2020, Tang *et al*., 2023, Tang *et al*., 2024) Here, “connectomic” refers to the use of information from an individual’s spatial neuronal connections, known as the connectome, to guide rTMS targeting. These targets are “parcel-guided,” meaning that the targets are based on parcellations from a multi-modal cortical map of the brain created by Glasser *et al*. 2016 from myelin mapping, rsfMRI, task-based fMRI, and topographical organisation of the brain (Glasser *et al*., 2016). This method leverages the precision of surface-based multimodal parcellation schemes, which not only enhance reproducibility across studies but also refine neuromodulatory targeting (Moreno-Ortega *et al*., 2020). Given that even millimetre-scale differences between parcels can distinctly influence network connectivity, precise targeting is critical to ensuring the intended therapeutic effects while minimising unintended modulation (Rosen *et al*., 2021).

### Neuronavigation

Applying a personalised and connectome-based approach resulted in targets across the entire brain. The Localite TMS Navigator (Bonn, Germany) neuro-navigation system was used to track the positions of the patients’ heads and the TMS coil, providing real-time feedback on the coil’s location over cortical targets displayed on T1 images. This allowed precision placement of the TMS coil and motion monitoring during stimulation.

### rTMS treatment

Prior to rTMS treatment, the resting motor threshold was determined for each patient. This threshold was determined as the minimum effective stimulation intensity to provoke an observable motor response in the left or right hand. If patients had fluctuations in factors that are known to modulate cortical excitability, such as sleep quality, alcohol intake, caffeine intake, or prescription medication use, the RMT would be re-tested daily. Stimulation was delivered at 80% of the RMT.

For each patient a maximum of 3 personalised targets was prescribed. Potential targets were excluded if they were located deeper than 35 mm from the head’s surface as they were considered beyond the effective field strength of the Magventure Cool-365 butterfly coil (Alfaretta, USA) (Deng *et al*., 2013). Stimulation intensity was slowly ramped to 80% RMT in the first sessions to ensure tolerability.

Each target received an accelerated theta burst stimulation (aTBS) protocol, either intermittent or continuous, consisting of 25 neuro-navigated TBS sessions, 5 per day over 5 days, either consecutively or spaced over 10 days. Each session lasted approximately 15 minutes, and between stimulations there was a 45- to 60-minute window.

cTBS was administered as one train of 600 stimuli applied in 50-Hz triplet pulses every 200 ms at 5 Hz, totalling 1800 pulses over 2 minutes. iTBS was initially administered as 40 trains of 10 stimuli applied in 50-Hz triplet pulses every 200 ms at 5 Hz with an intertrain interval of 6.3s. However, in 2021 the iTBS protocol was updated to reflect the exact protocol that received FDA clearance for MDD in 2018: 20 trains of 10 stimuli applied in 50-Hz triplet pulses every 200 ms at 5 Hz with an intertrain interval of 8 s, for a total of 600 pulses.

### Safety protocol

To ensure patient safety and minimise seizure risk during treatment, patients were administered the minimum effective dosage known as the RMT. If more than two days passed between treatments, the RMT was automatically retested. All technical staff were trained in seizure management and first aid. Earplugs were recommended, particularly for those with hyperacusis, tinnitus, or stimulation sites near the ears due to background noise of the machine. Maintaining hydration during treatment was also encouraged. Any adverse side effects were recorded by technicians administering TMS.

The Common Terminology Criteria for Adverse Events (CTCAE) five-grade severity scale was used to classify and monitor adverse side effects. Common TMS-related symptoms, such as transient headaches, scalp discomfort, and facial twitching, were classified as mild (Grade 1) when they required no medical intervention. Symptoms requiring medication, such as persistent headaches or discomfort, were categorised as moderate (Grade 2). A tonic-clonic seizure, should it have occurred, would be categorized as severe (Grade 3), medically significant but not life threatening. Grades 4 and 5 corresponded to life threatening and fatal adverse events respectively. This classification approach aligns with established clinical research standards on the safety of TMS, including TBS, ensuring comparability with existing literature.

## Results

### Patient demographics

The patient demographics are described in Table 1 with the top six most common treated conditions. Additional diagnoses and symptoms can be found in Supplement 1. Within this dataset, 165 individual patients were treated with parcel-guided rTMS and 30 patients returned for one or more rTMS retreatments. In total, there were 202 target sets. There were 578 total target parcellations across this patient population with 92 unique parcellations (left and right side counting as separate parcellations).

### Overall side effects

After rTMS, common side effects included fatigue (102/202, 50%), local muscle twitching (89/202, 43%), headaches (49/202, 23%), and discomfort (31/202, 17%).

Only mild (Grade 1) adverse side effects were reported.

The top 10 unique parcellations that were commonly found in the target sets included L8av (52%), LPGs (28%), LTe1m (21%), RTe1m (18%), LPFM (17%), Ls6–8 (13%), Rs6–8 (9%), L46 (7%), L1 (6%), and L6v (6%) (Fig. 2A and B). Among the target sets that contained at least one of the regions mentioned above, the percentage (out of the total number of times this region was part of a target set) of patients who reported headaches ranged from were 14 – 47%, fatigue was 35 – 67%, and local muscle twitching were 20 – 85% (Fig. 2C). Area Te1m in both the left and right side had the highest percentage of muscle twitching (72 and 85% respectively).

Next, we identified the parcellations from target sets with the highest rate of headaches, fatigue, and local muscle twitching. Parcellations targeted in at least 4 target sets were included in this analysis. Of the 4 times that LPHT was targeted, 4 patients reported headaches (4/4, 100%). Headaches were also common to target sets containing LPSL (3/4, 75%), R8c (3/6, 50%) and L46 (7/15, 47%). Fatigue was most common in patients with target sets containing to R6v (6/6, 100%), L8c (5/5, 100%), R4 (4/5, 80%), R55b (4/5, 80%), LTGv (3/4, 75%), and R1 (6/8, 75%). Muscle twitching was observed in target sets with targets to RTGv (5/5, 100%), LTGv (4/4, 100%), RTe1m (33/39, 85%), and LTe1m (32/45, 71%).

A few individuals reported discomfort following rTMS to specific parcellations: four patients reported discomfort to the RTE1m and one patient each reported discomfort to the right cerebellum, LTE1m, LSTDa, L6v, and L55b.

### Side effects when targeting parcellations within the dlPFC

There are 13 parcellations within the dlPFC: 8C, 8Av, i6-8, s6-8, SFL, 8BL, 9p, 9a, 8Ad, p9-46v, a9-46v, 46, and 9-46d. Out of the 578 total targets within this cohort, 212 were within the dlPFC (36%). The number of patients with these targets and the percentage of patients who experienced side effects to rTMS are shown in Table 2. L8Av was the most targeted region within the dlPFC, and the most reported side effect in target sets containing this parcellation was fatigue (49/110, 45%).

**Table 2.** Common targets within the dlPFC. Number of target sets containing the 13 parcellations of the dlPFC (Columns 3 and 5). Number of patients who experienced side effects to stimulating target sets that contain those regions (Columns 4 and 6). The names of locations are taken from Glasser et al 2016

1. Parcellation	2. Whole Name	3. Left Target: Number of target sets	4. Left Target: Number of patients with Headache, Fatigue, and Muscle Twitching (% out of total target sets with that target)	5. Right Target: Number of target sets	6. Right Target: Number of patients with Headache, Fatigue, and Muscle Twitching (% out of total target sets with that target)
8C	Area 8C	5	Headache: 2 (40) Fatigue: 5 (100) Muscle Twitching: 3 (60)	6	3 (50) 3 (50) 3 (50)
8Av	Area 8Av	110	21 (19) 49 (45) 38 (35)	1	0 (0) 0 (0) 1 (100)
i6-8	Inferior 6-8 Transitional Area	0	0 (0) 0 (0) 0 (0)	1	0 (0) 0 (0) 1 (100)
s6-8	Superior 6-8 Transitional Area	28	11 (39) 14 (50) 8 (29)	20	3 (15) 11 (55) 4 (20)
SFL	Superior Frontal Language Area	1	0 (0) 0 (0) 1 (100)	10	2 (20) 5 (50) 4 (40)
8BL	Area 8B Lateral	1	0 (0) 0 (0) 0 (0)	0	0 (0) 0 (0) 0 (0)
9p	Area 9 Posterior	0	0 (0) 0 (0) 0 (0)	0	0 (0) 0 (0) 0 (0)
9a	Area 9 Anterior	0	0 (0) 0 (0) 0 (0)	0	0 (0) 0 (0) 0 (0)
8Ad	Area 8Ad	0	0 (0) 0 (0) 0 (0)	1	1 (100) 1 (100) 0 (0)
a9-46v	Area Anterior 9-46v	4	0 (0) 2 (50) 1 (25)	2	1 (50) 0 (0) 1 (50)
p9-46v	Area Posterior 9-46v	2	2 (100) 1 (50) 1 (50)	1	0 (0) 1 (100) 1 (100)
46	Area 46	15	7 (47) 8 (53) 5 (33)	3	1 (33) 2 (67) 1 (33)
9-46d	Area 9-46d	0	0 (0) 0 (0) 0 (0)	1	1 (100) 0 (0) 0 (0)

### Side effects when targeting parcellations outside of the dlPFC

**Figure 3.**
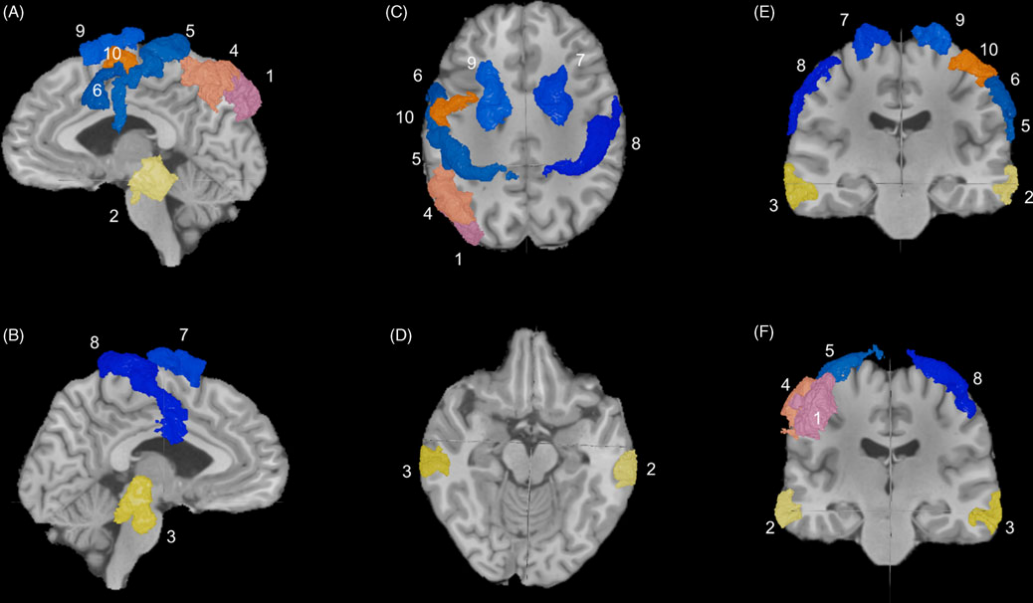
The most common stimulated parcels outside of the dlPFC. This figure depicts the location of the most common parcels stimulated that lie outside of the dlPFC in order of frequency. Locations are laid over standardized anatomical images. From most to least common 1. LPGs, 2. LTe1m, 3. RTe1m, 4. LPFM, 5. L1, 6. L6v, 7. R6ma, 8. R1, 9. L6Ma, 10. L55b. a) left sagittal, b) right sagittal, c) top axial, d) bottom axial, e) front coronal, f) back coronal.

366 of the 578 total target parcellations were outside of the dlPFC (63%). These were 74 unique parcellations. The top ten most frequently targeted regions were LPGs, RTe1m, LTe1m, LPFM, L1, L6v, R6ma, L6ma, L55b, and R1 (Fig. 3). The number of patients with these targets and the percentage of patients who experienced side effects to rTMS are shown in Table 3.

**Table 3.** Common targets outside of the dlPFC. Number of target sets that contain these common parcellations outside of the dlPFC (Columns 4 and 6). Number of patients who experienced side effects to stimulating target sets that contain those regions (Columns 5 and 7)

1. Parcellation	2. Whole Name	3. Location	4. Left Target: Number of target sets	5. Left Target: Number of patients with Headache, Fatigue, and Muscle Twitching (% out of total target sets)	6. Right Target: Number of target sets	7. Right Target: Number of patients with Headache, Fatigue, and Muscle Twitching (% out of total target sets)
PGs	Area PGs	Inferior Parietal Cortex	60	13 (22) 21 (35) 18 (30)	3	0 (0) 2 (67) 1 (33)
Te1m	Area TE1 Middle	Lateral Temporal Cortex	45	15 (33) 26 (58) 32 (71)	39	12 (31) 18 (46) 33 (85)
PFM	Are PFm Complex	Inferior Parietal Cortex	37	10 (27) 16 (43) 16 (43)	7	3 (43) 3 (43) 1 (14)
1	Area 1	Primary Somatosensory Cortex	14	2 (14) 9 (64) 6 (43)	8	2 (25) 6 (75) 3 (38)
6v	Ventral Area 6	Premotor Cortex	12	2 (17) 8 (67) 6 (50)	6	1 (16) 6 (100) 3 (50)
6ma	Area 6 m Anterior	Supplementary Motor Areas	8	1 (13) 4 (50) 1 (13)	10	1 (10) 4 (40) 5 (50)
55b	Area 55b	Posterior Middle Frontal Gyrus	8	0 (0) 4 (50) 5 (63)	5	0 (0) 4 (80) 2 (40)
PHT	Area PHT	Lateral Temporal Cortex	4	4 (100) 2 (50) 0 (0)	0	0 (0) 0 (0) 0 (0)

### Adverse side effects and other less common side effects

No adverse side effects such as seizures were observed in any of the patients within this cohort. Only three patients discontinued rTMS to one parcellation due to discomfort (but continued rTMS in other regions). These patients had different targets: RTE1m, LTE1m, and LTGv.

Other less common side effects included staring spells which were observed in 3 patients who previously reported experiencing such events pre-treatment. Five patients reported feeling nausea. Three patients reported dizziness. Two patients had nosebleeds.

### Side effects in vulnerable populations

In our patient cohort, 15 patients had craniotomies and 11 patients had strokes prior to presenting to our clinic. Traditionally, these patients would be contraindicated for rTMS due to concerns about this population with an increased seizure risk and previously limited treatment data. All vulnerable populations were properly consented and informed of the potential risks of TMS as related totheir specific conditions. Of note, some patients returned for additional rTMS retreatment, so any side effects would be reported per target set.

Within the post-stroke subpopulation, one patient returned for 2 additional rTMS treatments and another patient returned for 1 additional rTMS treatment for a total of 14 target sets. There were 9/14 (64%) instances of fatigue, 6/14 (43%) instances of twitching, 1/14 (6%) instances of discomfort, and no reported headaches.

Within the post-craniotomy population, two patients returned for 1 additional rTMS treatment each for a total of 17 target sets. There were 3/17 (18%) instances of headaches, 14/17 (82%) instances of fatigue, 7/17 (41%) instances of twitching, 5/17 (29%) instances of discomfort, and 2/17 (12%) cases of nausea.

## Discussion

This study aimed to retrospectively analyse the safety and tolerability of a personalised TMS protocol that utilises connectomics, stereotactic neuronavigation, and TBS to stimulate cortical targets across the brain. This is a large TMS patient dataset of 165 patients and 92 unique parcellation targets. The main finding of this study was that the most common side effects were analogous to that of conventional rTMS approaches: headache, fatigue, muscle twitching, and discomfort. Safety profiles were reported for the 13 parcellations within the dlPFC. This study also reports the safety profiles of targeting regions outside of the conventional dlPFC, including the inferior parietal cortex, lateral temporal cortex, primary somatosensory cortex, premotor cortex, supplementary cortex, and posterior middle frontal gyrus.

This study retrospectively analysed the safety and tolerability of a personalised rTMS protocol that integrates individualized connectomic data, stereotactic neuronavigation, and TBS to stimulate cortical targets across the brain. The findings indicate that this targeted approach maintains a safety profile comparable to conventional rTMS, with common side effects including headache, fatigue, muscle twitching, and discomfort. Importantly, this study provides novel insights into the tolerability of targeting regions beyond the dlPFC, including the inferior parietal cortex, lateral temporal cortex, primary somatosensory cortex, premotor cortex, supplementary cortex, and posterior middle frontal gyrus. This in turn may allow for more tailored and anatomically specific TMS approaches beyond traditional targets within a significant safety margin. In general, the side effects reported, particularly in Table 3, are minor and short-lived. Knowledge of the percent of patients experiencing these side effects at specific locations could aid in educating and guiding patients to understand potential side effects of their treatment.

### Comparison to other reports in literature

In comparison to other rTMS studies, we report a dropout rate of 0% and a target discontinuation rate of 1.8%, which is similar to the 2.5% discontinuation rate due to adverse effects estimated in a meta-analysis (Zis *et al*., 2020). This demonstrates the feasibility of our personalised connectomic TMS targeting approach which does not introduce new significant logistical constraints that can trouble large trials. Another study by O’Reardon et al., (2007) reported a higher discontinuation rate of 4.5% due to scalp discomfort and pain, which is comparable to the reason for discontinuation in the three patients from our study (O’Reardon *et al*., 2007). These findings suggest that discomfort remains a key factor in target discontinuation across rTMS studies, even when using personalised targeting approaches.

The occurrence of fatigue and local muscle twitching in this study is consistent with prior findings on mild adverse effects (MAEs). One study reported that more than 40% of TMS participants experience MAEs (Machii *et al*., 2006), which aligns with our observed rates. While headache was not the most common side effect in our study, the 28% of patients reporting headaches is consistent with the findings of another study (Loo *et al*., 2008). However, the incidence of discomfort (17%) in our cohort was substantially lower than the 39% reported by that group which may be due to the use of TBS, as lower treatment doses (80% RMT vs. 120% RMT) have been associated with reduced discomfort (Oberman *et al*., 2011).

Despite the overall reduction in discomfort, the incidence of TBS-associated MAEs in this study exceeded the ∼ 5% previously reported by Oberman *et al*. (2011). This discrepancy may be explained by our sample population containing a higher proportion of occipital, temporal, and parietal targets, which are regions associated with increased discomfort. These findings underscore the importance of target selection in mitigating discomfort when employing personalised rTMS protocols.

While these large-scale averages provide useful context for understanding general tolerability trends, the personalised nature of our protocol and inter-patient variability in target selection limit the generalisability of these figures. The variation in side effects across unique target sets, particularly those outside of the dlPFC, is of greater clinical interest.

For instance, 85% and 71% of the target sets containing LTE1m and RTE1m, respectively, resulted in muscle twitching. This is a marked increase compared to the 44% muscle twitching incidence observed by stimulating temporal areas at 110% MT(Loo *et al*., 2010). This difference is likely due to the anterior placement of TE1m, as more posterior and medial temporal sites tend to have fewer superficial muscle fibres available for stimulation (Loo *et al*., 2008). These findings suggest that targeting anterior temporal areas may be associated with a higher incidence of muscle twitching and should be considered when selecting stimulation sites for individualised treatment plans.

A study that used fMRI-guided neuronavigation to target the left inferior parietal cortex for the treatment of Alzheimer’s disease reported a discontinuation rate of ∼ 8% due to discomfort or transient fatigue (Jia *et al*., 2021). In our study, parcellations PGs and PFM within the inferior parietal cortex were well tolerated, with fatigue (35–43%) and muscle twitching (30–43%) remaining within expected ranges. Variability in tolerability between studies may be due to differences in target selection methodologies, session numbers, and stimulation intensity.

### Vulnerable patient cohorts

Another consideration is that TMS has been previously contraindicated by the presence of brain lesions based on evidence that stimulation too close to these sites could induce seizure, thereby limiting treatment options for patients recovering from craniotomies or stroke (Rossi *et al*., 2021, Cogne *et al*., 2017, O’Neal *et al*., 2020).

There is notable level II evidence supporting the use of rTMS for motor recovery in stroke patients (Khedr *et al*., 2005, Du *et al*., 2016) and post-surgical glioma patients (Ille *et al*., 2021, Rosenstock *et al*., 2025), demonstrating significant functional improvements compared to sham treatments. The presence of both neurosurgical and stroke patients within our study sample and within previous studies applying the same approach (Yeung *et al*., 2021, Tang *et al*., 2022, Dadario *et al*., 2022) without the occurrence of moderate or serious adverse events supports a growing body of evidence that neuronavigation utilising personalised brain maps is an effective way that stimulation can be delivered while avoiding these lesional regions, and that these patient cohorts may not differ significantly in SAE likelihood compared to more standard ones (Caulfield *et al*., 2022, O’Neal *et al*., 2020, Yeung *et al*., 2021).

### Limitations

The main limitation to this paper was the lack of a control group, as there is evidence of pronounced placebo and nocebo effects associated with TMS (Zis *et al*., 2020). Additionally, there is high heterogeneity in how TMS is administered between clinics, as 100 or 120% RMT may be used during treatment, and RMT can be substituted for active motor threshold (AMT) which typically yields lower values (Temesi *et al*., 2014). Moreover, the impact that coil geometry (i.e. figure of 8 versus butterfly) has on stimulation depth and field dispersion suggests that the incidence of adverse effects could be altered by coil selection (Maizey *et al*., 2013).

Another limitation is the software’s dataset of only 200 controls which may be considered limited given the complexity of the brain disorders treated and the range of demographics within condition types. This dataset may lack sufficient statistical power to detect subtle abnormalities across a diverse patient population. Future studies with larger, multi-site cohorts and broader demographic representation will be crucial to validating and extending these findings. As of 2024 this dataset has been updated to 2500 controls using the OpenNeuro and SchizConnect datasets as well as a mix of controls from the UK Biobank, the Brain Genomics Superstruct Project (BGSP) and the Amsterdam Open MRI Collection (AOMIC). As the control dataset is updated over time, the software’s anomaly detection will improve for future patients.

The large size in patient numbers and target selections in this study may benefit patients undergoing personalised TMS as patients are made aware of the likelihood of specific side effects related to their targets. However, caution should be exercised when interpreting the side effect profile for regions with limited patient data, particularly those that are infrequently represented in this dataset. Moreover, the side effects in this study were reported for target sets with two or three parcellation selections rather than individual parcellation targets. Due to the individualised nature of this study paradigm, patients do not have the exact same target sets as each other. Therefore, one cannot directly relate one parcellation to its side effects, per say, from this study. Larger amounts of patient data preferably in sham-controlled studies should be established in the future to verify the percentage of side effects in each parcellation. Nevertheless, these current results could improve the process of informed consent and initial target selection when planning personalised treatment.

## Conclusion

Individualised, parcel-guided rTMS to regions within and outside of the dlPFC is safe with no adverse, long-lasting effects and has similar side effect profiles as those reported in literature. rTMS targeting to various brain regions in different disease states is well tolerated.

## Supporting information

Tang et al. supplementary material Tang et al. supplementary material

## Data Availability

Data availability upon request.

## Supplementary material

The supplementary material for this article can be found at https://doi.org/10.1017/neu.2025.9.
