# Supplementary material for: Evaluating the safety profile of connectome-based repetitive transcranial magnetic stimulation
Source: Acta Neuropsychiatr. 2025 Mar 21;37:e61. doi: 10.1017/neu.2025.9 (PMC13130353; doi:10.1017/neu.2025.9)
Supplement: Tang et al. supplementary material [file S0924270825000092sup001.docx]

Supplement 1: Other conditions in the patient population. Note that the total does not add up to 100% because patients may have multiple symptoms/diagnoses.

| **Conditions** | **Number of Patients** | **% (out of 165 patients)** |
| --- | --- | --- |
| Anxiety | 65 | 39 |
| Depression | 58 | 35 |
| TBI | 26 | 16 |
| Cognition (Excluding Alzheimer’s Disease/Dementia) | 18 | 11 |
| Post-surgical rehabilitation | 15 | 9 |
| Stroke | 11 | 7 |
| Migraine/Headaches | 11 | 7 |
| Pain | 8 | 5 |
| PTSD | 8 | 5 |
| Tinnitus | 7 | 4 |
| OCD | 6 | 4 |
| Vertigo | 5 | 3 |
| Parkinson's | 5 | 3 |
| Alzheimer's Disease/Dementia | 5 | 3 |
| Addiction | 3 | 2 |
| ADHD | 3 | 2 |
| Fatigue | 3 | 2 |
| Ataxia | 2 | 1 |
| Dysphagia | 2 | 1 |
| Anorexia | 2 | 1 |
| Friedrich's Ataxia | 2 | 1 |
| Mobility | 2 | 1 |
| Sleep | 2 | 1 |
| Visual and/or Auditory Hallucinations | 2 | 1 |
| Autism | 1 | 1 |
| Hypobulia | 1 | 1 |
| Multiple Sclerosis | 1 | 1 |
| Photophobia | 1 | 1 |
| Motor Neurone Disease | 1 | 1 |
| Spasmodic Dysphonia | 1 | 1 |
| Phobia | 1 | 1 |
| Dyslexia | 1 | 1 |
| Hemiplegia | 1 | 1 |
| Hypersensitivity | 1 | 1 |
| Stress | 1 | 1 |
| Fibromyalgia | 1 | 1 |
| Panic | 1 | 1 |
| Balance | 1 | 1 |
